# Supplementary material for: Causal Relationship between Aging and Anorexia Nervosa: A White-Matter-Microstructure-Mediated Mendelian Randomization Analysis
Source: Biomedicines. 2024 Aug 16;12(8):1874. doi: 10.3390/biomedicines12081874 (PMC11351342; doi:10.3390/biomedicines12081874)
Supplement: Supplementary file 1 [file biomedicines-12-01874-s001.zip › Supplementary Figure/Figure S4/Singe SNP plot of MR analysis for TL to ubm-b-1683.pdf]

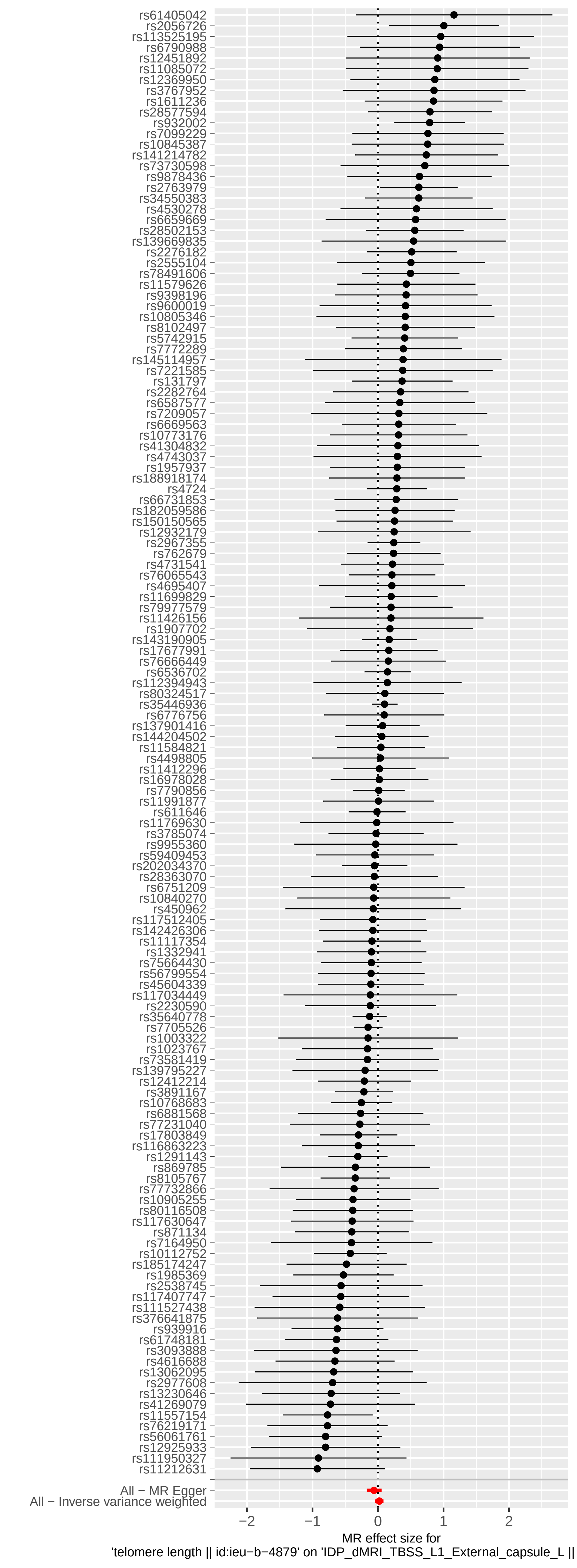
rs61405042
rs2056726
rs113525195
rs6790988
rs12451892
rs11085072
rs12369950
rs3767952
rs1611236
rs28577594
rs932002
rs7099229
rs10845387
rs141214782
rs73730598
rs9878436
rs2763979
rs34550383
rs4530278
rs6659669
rs28502153
rs139669835
rs2276182
rs2555104
rs78491606
rs11579626
rs9398196
rs9600019
rs10805346
rs8102497
rs5742915
rs7772289
rs145114957
rs7221585
rs131797
rs2282764
rs6587577
rs7209057
rs6669563
rs10773176
rs41304832
rs4743037
rs1957937
rs188918174
rs4724
rs66731853
rs182059586
rs150150565
rs12932179
rs2967355
rs762679
rs4731541
rs76065543
rs4695407
rs11699829
rs79977579
rs11426156
rs1907702
rs143190905
rs17677991
rs76666449
rs6536702
rs112394943
rs80324517
rs35446936
rs6776756
rs137901416
rs144204502
rs11584821
rs4498805
rs11412296
rs16978028
rs7790856
rs11991877
rs611646
rs11769630
rs3785074
rs9955360
rs59409453
rs202034370
rs28363070
rs6751209
rs10840270
rs450962
rs117512405
rs142426306
rs11117354
rs1332941
rs75664430
rs56799554
rs45604339
rs117034449
rs2230590
rs35640778
rs7705526
rs1003322
rs1023767
rs73581419
rs139795227
rs12412214
rs3891167
rs10768683
rs6881568
rs77231040
rs17803849
rs116863223
rs1291143
rs869785
rs8105767
rs77732866
rs10905255
rs80116508
rs117630647
rs871134
rs7164950
rs10112752
rs185174247
rs1985369
rs2538745
rs117407747
rs111527438
rs376641875
rs939916
rs61748181
rs3093888
rs4616688
rs13062095
rs2977608
rs13230646
rs41269079
rs11557154
rs76219171
rs56061761
rs12925933
rs111950327
rs11212631
All − MR Egger
All − Inverse variance weighted
−2
−1
0
1
2
MR effect size for
'telomere length || id:ieu−b−4879' on 'IDP_dMRI_TBSS_L1_External_capsule_L ||
